# Changes in Muscle Power and Muscle Morphology with Different Volumes of Fast Eccentric Half-Squats

**DOI:** 10.3390/sports7070164

**Published:** 2019-07-05

**Authors:** Evangelia Zacharia, Polyxeni Spiliopoulou, Spyridon Methenitis, Angeliki-Nikoletta Stasinaki, Nikolaos Zaras, Constantinos Papadopoulos, Giorgos Papadimas, Giorgos Karampatsos, Gregory C. Bogdanis, Gerasimos Terzis

**Affiliations:** 1 Sports Performance Laboratory, School of Physical Education and Sport Science, National and Kapodistrian University of Athens, Daphne, 17237 Athens, Greece; 2 Department of Life and Health Sciences, School of Sciences and Engineering, University of Nicosia, CY-1700 Nicosia, Cyprus; 3 A’ Neurology Clinic, Aiginition Hospital, Medical School, National and Kapodistrian University of Athens, 11528 Athens, Greece

**Keywords:** eccentric training, fiber type composition, females, ultrasound, muscle hypertrophy

## Abstract

The aim of the study was to evaluate power performance and muscle morphology adaptations in response to 5 weeks of fast-eccentric squat training (FEST) performed twice per week, with three different training volumes. Twenty-five moderately trained females were assigned into three groups performing eight repetitions of FEST of either four sets (4 × 8 group; N = 9), 6 sets (6 × 8 group; N = 8) or eight sets (8 × 8 group, N = 8). Before and after the intervention, countermovement jumping height (CMJh) and power (CMJp), half squat maximal strength (1-RM), quadriceps cross-sectional area (QCSA) and vastus lateralis (VL) architecture and fiber type composition were evaluated. Significant increases (p < 0.05) were found for all groups, with no differences among them in 1-RM (4 × 8: 14.8 ± 8.2%, 6 × 8: 13.1 ± 9.2% and 8 × 8: 21.6 ± 7.0%), CMJh (4 × 8: 12.5 ± 8.5%, 6 × 8: 11.3 ± 9.3% and 8 × 8: 7.0 ± 6.2%), CMJp (4 × 8: 9.1 ± 6.0%, 6 × 8: 7.1 ± 5.2% and 8 × 8: 5.0 ± 3.9%) and QCSA (4 × 8: 7.7 ± 4.7%, 6 × 8: 9.0 ± 6.8% and 8 × 8: 8.2 ± 6.5%). Muscle fiber type distribution remained unaltered after training in all groups. VL fascicle length increased and fascicle angle decreased only in 6 × 8 and 8 × 8 groups. In conclusion, four sets of eight fast-eccentric squats/week increase lower body power and strength performance and maintain type IIX muscle fibers after 5 weeks, at least in moderately trained females.

## 1. Introduction

Muscle power determines the ability to perform explosive actions both in sports and in everyday life. Resistance training performed with maximum concentric velocity is an effective intervention to enhance muscle power [1,2]. Interestingly, increases in power performance have been observed both after high and lower volumes of explosive resistance training. For example, Kyrolainen et al. [3] found a 23% increase in jumping power after progressively increasing the weekly explosive actions to 180 per session. Other studies reported similar increases in explosive performance with 100–300 repetitions per session [3,4,5,6,7,8]. Then again, jumping power was increased 11% after power training with less than 80 repetitions per training session [7,9,10,11,12,13]. Yet, it is of practical importance to know whether even lower training volumes of explosive resistance training applied over short periods, may result in meaningful increases in lower body muscle power. 

Power resistance training involves primarily high-velocity movements with body weight or with additional external loads, e.g., jumps [3], ballistic movements [7], eccentric actions [11,14] or a combination of the above [9,12]. The ability for high muscle power production seems to depend on many biological parameters, including muscle mass, muscle architecture, muscle fiber type composition neural activation and rate of force development [4,5,11,15,16,17,18,19,20,21,22]. Effective power training programs promote at least some of these neuromuscular adaptations. Specifically, increases in explosive performance in response to chronic power training have been observed concomitantly with the preservation of the proportion of type IIX muscle fibers [23,24] and significant increases in the cross-sectional area (CSA) of type II muscle fibers [9,23,25,26,27]. Moreover, muscle power increases have been linked with increases in muscle fascicle length [14] as well as increases in the number of activated motor units (MU), lower recruitment threshold of fast fibers, and greater discharge rates of these MUs, among other adaptations [4,28,29,30]. However, until now the effect of different power training volumes on muscle fiber composition and muscle architecture has never been investigated.

Among other resistance training regimens, eccentric training has stimulated the interest of sports scientists towards increasing explosive performance. This type of training exploits the ability of the muscle to produce greater maximum forces during the eccentric phase of action [31], and it seems to be a promising alternative method to induce significant neuromuscular adaptations leading to rapid increases in muscle power production [9,12,13,32,33,34,35,36,37]. Furthermore, it seems that the movement velocity during eccentric training may be an important element of the eccentric training stimulus contributing to muscle power enhancement [11,14]. Specifically, squat eccentric training with relative fast movements, either performed exclusively or combined with jumping exercises has been shown to increase explosive performance after a few weeks of training [9,11,12]. Interestingly, the increase in power performance with exclusive fast eccentric half squats has been linked to increases in vastus lateralis fascicle length [11]. However, the training volume applied in that recent study was relatively large, that is 162 repetitions per week applied for 6 weeks. Considering the efficacy of this type of training in increasing power performance, we aimed to explore whether a relatively lower volume of fast eccentric half-squats applied over a shorter period might also induce explosive performance enhancements. Therefore, aim of the present study was to investigate power performance and muscle adaptations after 5 weeks of fast-eccentric half-squats performed with three different training volumes in moderately trained females. It was hypothesized that all three training volumes would induce improvements in lower body explosive performance but that the higher training volume would lead to higher improvements in muscle power, compared with the lower training volume. In the present study, only female participants were recruited. This was decided due to the relative paucity of data regarding power training adaptations in female participants. Moreover, similar muscle morphology adaptations have been reported for males and females (or ever stronger for females) after the first weeks of resistance training [38], like that used in the present study.

## 2. Materials and Methods

### 2.1. Experimental Approach to the Problem 

Three groups of young, female, physical education students, with minimal resistance training experience, were trained for 5 weeks with either four, six, or eight sets, of eight repetitions of fast eccentric-only half squats. Before and after the training period, lower body strength and power performance as well as vastus lateralis muscle morphology and architecture were evaluated. After recruitment of the participants, before any other testing, anthropometric characteristics and lower extremity dominance (revised Waterloo Footedness Questionnaire, ICC = 0.92) were assessed. Counter movement jumping performance (CMJ) and half-squat maximum strength (1-RM) in a Smith machine (Smith Machine, Super Sport, Chaidari, Greece) were tested in the same day, one week later. Muscle architecture was assessed with ultrasonography and muscle biopsies were obtained from the same area of non-dominant vastus lateralis, three days later. After concluding these evaluations, each participant performed two training familiarization sessions, with 1-day rest between them. The training program included fast eccentric-only half squats in a Smith machine (see “Training” section). Participants completed a 5–week intervention, twice a week with at least 72 h rest between the training sessions. Finally, all the initial evaluations (T1) were repeated with the same order at least 1 week after the end of the last training session (T2).

### 2.2. Participants

Participants were recruited via advertisements at the local university. Responders visited the laboratory and completed a weekly recall self-reported physical activity questionnaire. The inclusion criteria for participants were: 1) female gender; 2) absence of systematic exercise training at least during the previous 12 months; 3) weight stability (±2 kg) for three months prior to entry; 4) absence of restraining orthopaedic/neuromuscular maladies; 5) age range between 18 to 27 years; and 6) absence of drug abuse or medications which are known to affect the neuromuscular system. Finally, twenty-five healthy female physical education students, gave their written consent to participate in the study, after being informed of the experimental procedures. They were assigned into three groups matched for non-dominant lower extremity vastus lateralis’ fascicle length and half-squat 1-RM (no differences among groups; p > 0.05). Basic anthropometric characteristics for the three groups are presented in Table 1. All procedures were in accordance with the Declaration of Helsinki and approved by the local university ethics committee (reference number 1039/14-02-2018), while all participants signed an informed consent before entering the study.

### 2.3. Procedures

#### 2.3.1. Training

All participants trained twice per week for 5 weeks, with at least 72 hours between sessions. Each group performed 8 repetitions of eccentric-only half squats in each set, but different number of sets in accordance to the respective training group: 4 × 8, 6 × 8, or 8 × 8 (sets × repetitions) with 3 minutes rest between sets. Each training session was initiated with a 5-minute warm-up on a stationary bicycle at 50–75 Watts and static stretching for the muscles of the lower extremities. Each participant performed the downward movement only (eccentric contraction) of the half-squat with instructions to lower the load as fast as possible, in a controlled movement and stop when knees reached an angle of 90°, with a rapid isometric contraction [9,11,12]. Then participants placed the barbell on a block which was placed 1 cm below, to stop the downward movement of the barbell. The duration of each repetition was between 0.7 to 1.1 s, as previously described [9]. The ascent of the bar to the initial position was performed with an electric motor and the next repetition was performed 10 seconds after the completion of the previous repetition. After the initial measurements and before the 5-week training period, all participants underwent two familiarization sessions. During these sessions, all participants were familiarized with the fast-eccentric exercise. A mirror was placed 3 meters in front of the Smith machine to help participants achieve the correct exercise form, while both in familiarization and training sessions, a metronome was used, to help participants keeping the rhythm/speed of the downward movement of Squatting. The external loads during the two familiarization sessions were set to 30% and 40% of the half-squat 1-RM, respectively [11]. For further information about the training stimuli used in the present study please refer to Bogdanis et al. [9], Stasinaki et al. [11] and Terzis et al. [12]. Familiarization and training sessions were performed in the same Smith machine. A Borg CR10 scale was used to obtain the rating of perceived exertion (RPE) of the training load, after the end of each training session. During the first week of training, the external load was set at 50% of the initial half-squat 1-RM, and thereafter it was increased to 55%, 60%, 65%, and 70% of the initial half-squat 1-RM, for each successive week. 

#### 2.3.2. Jumping Performance

Participants warmed-up for 5 minutes on a stationary bicycle at 50 Watt before performing three CMJs with submaximal intensity. Subsequently, they performed 3threemaximal CMJs with arms akimbo, with 2 min rest between each jump, on a force platform (WP1000kg weighting platform, Applied Measurements Ltd Co. UK, sampling frequency 1 kHz) as previously described [16,17,20]). Force platform data were recorded and analyzed (Kyowa sensor interface PCD-320A) to calculate the following variables [16,39]: Jump height (cm) = ((0.5 × flight time)^2^ × 2^−1^) × 9.81 and Maximum Power (W) = (body weight + Fmax) × 9.81 × flight time. The signal was filtered using a fourth order low pass Butterworth filter with a cutoff frequency of 10 Hz. The best performance in jump height was used for further analysis. The ICCs for jump height and power were 0.87, (95% CI: Lower = 0.83, Upper = 0.95) and 0.91 (95% CI: Lower = 0.90, Upper = 0.99), respectively, n = 13. 

#### 2.3.3. RM Half-Squat Strength Evaluation

Maximal half-squat strength was assessed in a Smith machine. Briefly, 10 minutes after the CMJ testing, participants performed 2–3 half-squat sets of 6–8 repetitions with increasing loads. Subsequently, they performed single repetition sets with incremental loads, with 3-minute rest between sets, until they were unable to lift a heavier load. Knee bending was not allowed beyond 90°. An adjustable iron rack was placed in the Smith machine to restrict knee bending under 90°. In all cases, two of the authors were present and vocally encouraged the participants. The ICC for 1-RM testing has been previously determined in our laboratory (ICC = 0.92,) [7,8].

#### 2.3.4. Evaluation of Muscle Architecture 

Ultrasound images, both before and after the training intervention were obtained during the morning hours. B-mode axial-plane ultrasound measurement (Product model Z5, Shenzhen Mindray Bio-Medical Electronics Co., Ltd, Shenzhen, China) was performed with a 10 MHz linear-array probe (38-mm width). All ultrasound images were analyzed with image analysis software (Motic Images Plus, 2.0, Motic Europe, Barcelona, Spain). Ultrasound images were obtained from the non-dominant lower extremity, performing the Extended-Field-Of-View technique on vastus lateralis (architecture analysis) and on quadriceps (CSA analysis), as previously described [11,25,40]. Briefly, firstly panoramic ultrasound images were obtained longitudinal to the femur on vastus lateralis, at 50% of the distance from the central palpable point of the greater trochanter to the lateral condyle of the femur [11,41]. Two images were taken from each participant for the analysis of muscle thickness, pennation angle and fascicle length of vastus lateralis and the mean of the two were used for statistical analysis. Muscle thickness was defined as the distance between the superficial and deep aponeurosis and was analyzed at the exact point of 50% of the distance from the central palpable point of the greater trochanter to the lateral condyle of the femur. Fascicle angle was defined as the angle of insertion of muscle fascicles into the deep aponeurosis. Fascicle length was defined as the fascicular path between the insertions of the fascicle into the upper and deeper aponeurosis. For some individuals, there was a tendency for fascicles to curve near the superficial aponeurosis. In these cases, fascicle length was measured with a curved line tool [11]. The ICCs for these evaluations have been explored recently [11] and were 0.97 (95% CI: 0.87–0.99, p = 0.001) for muscle thickness, 0.88 (95% CI: 0.60-0.97, p = 0.001) for fascicle angle and 0.84 (95% CI: 0.47–0.96, p = 0.001) for fascicle length. 

Secondly, panoramic ultrasound images were obtained transversely on the femur, at the 40% of the distance (proximal to the knee) from the center of the patella to the medial aspect of the anterior superior iliac spine [40,41]. Each panoramic ultrasound image pictured the cross sectional area (CSA) of whole quadriceps with its four heads separately. Two images were taken from each participant for the analysis of the CSA of whole quadriceps femoris (Quad). The ICC for quadriceps CSA evaluation was 0.97 (95% CI: 0.87–0.99, p = 0.001; n = 10).

#### 2.3.5. Muscle Biopsies and Histochemistry

Muscle samples were obtained from vastus lateralis of the non-dominant lower extremity, with Bergström needles under local anesthesia. Initial (T1) samples were obtained 20 cm from mid patella while the final (T2) samples were obtained 5 cm proximally to the initial site. Samples were aligned, placed in embedding compound and frozen in isopentane pre-cooled to its freezing point, and subsequently stored in liquid nitrogen until analysis. Serial cross sections of 10 μm thickness were cut at −20 °C and stained for myofibrillar ATPase after pre incubation at pH 4.3, 4.6, and 10.3 [9,12,16,19,42,43]. A mean of 411 ± 188 at T1 and 496 ± 186 at T2 muscle fibers, from each participant were classified as type I, IIA, or IIX. The cross-sectional area (CSA) of all the classified muscle fibers was measured with an image analysis system (Image Pro, Media Cybemetics Ins, Silver Spring, MD, USA) at a known and calibrated magnification. The ICCs for the percentage of type I, IIa and IIX fibers in our laboratory is 0.96, 0.95 and 0.93, respectively (95% CI: Lower = 0.91, 0.92, 0.87 and Upper = 0.99, 0.98, 0.95, respectively).

### 2.4. Statistical Analyses 

A post hoc power analysis (G*Power ver 3.1; FrankFaul, Universitat Kiel, Germany), was performed according to the study design, the number of the participants and the lower Partial Eta Squared of the significant contrasts that were found, which revealed an actual power of 0.897 for the results of the present study. The assumption of normality was tested prior to any further statistical analysis, with the Shapiro–Wilk test (p > 0.05). All data are presented as mean and standard deviation (±SD). Two-way repeated measures analyses of variance [3 (groups) × 2 (time); ANOVAs]. When significant differences were found, Bonferroni Post-Hoc tests were used for the investigation of the differences of the absolute values among the three groups, before and after training. One-way ANOVAs followed by Bonferroni Post-Hoc tests were used to compare the percentage changes (%; pre- vs. post-training) among the three groups. Partial eta squared was also evaluated, while the magnitude of effect size (ES) for pairwise comparisons, was determined by Hedges’ g (small: <0.3, medium: 0.3–0.8, large: >0.8). Pearson’s product moment correlation coefficient was used to explore correlations between the changes in performance and biological parameters evaluated in the present study. Statistical analyses were performed with SPSS Statistics (Ver. 21, IBM Corporation, New York, USA). P ≤ 0.05 was used as a 2-tailed level of significance.

## 3. Results

**Figure 1:**
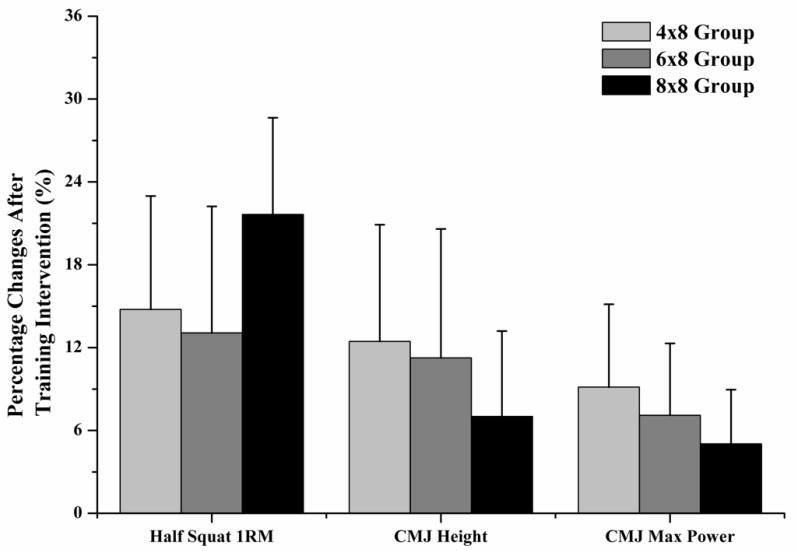
Percentage changes in half-squat maximum strength, countermovement jump (CMJ) height and maximum power after 5 weeks of training with fast eccentric-only half squats for each group.

No significant differences were found in the initial evaluations among the groups (p > 0.05). Half-squat 1RM increased significantly after the training intervention in all groups (Factor Time: p < 0.001; η^2^: 0.870). Specifically, it was increased 14.8 ± 8.2%, 13.1 ± 9.2% and 21.6 ± 7.0% for the 4 × 8 group (T1: 111.7 ± 17.1 kg, T2: 127.2 ± 14.1 kg; Hedges’ g = 1.096), the 6 × 8 group (T1: 109.4 ± 11.5 kg, T2: 123.1 ± 11.3 kg; Hedges’ g: 1.284) and the 8 × 8 group (T1: 113.1 ± 23.3 kg, T2: 136.6 ± 22.7 kg, Hedges’ g: 1.096). No significant differences were found for 1RM increases among the groups (Group, Time × Group Interaction: p > 0.05), although the percentage increase in the 8 × 8 group was higher (Figure 1). CMJ height was increased (Factor Time: p < 0.001; η^2^: 0.669) in all groups, with no significant differences among groups (Group, Time × Group Interaction: p > 0.05). Specifically, CMJ height increased by 12.5 ± 8.5%, 11.3 ± 9.3% and 7.0 ± 6.2% for the 4 × 8 group (T1: 23.8 ± 2.7 cm, T2: 26.8 ± 3.7 cm; Hedges’ g: 1.027), 6 × 8 group (T1: 22.3 ± 3.1 cm, T2: 24.7 ± 2.9 cm; Hedges’ g: 0.834) and 8 × 8 group, respectively (T1: 25.6 ± 3.6 cm T2: 27.5 ± 4.7 cm; Hedges’ g: 0.648; Figure 1). CMJ max power was increased significantly in all groups (Factor Time: p < 0.001; η^2^: 0.779), by 9.1 ± 6.0%, 7.1 ± 5.2% and 5.0 ± 3.9% for the 4 × 8 group (T1: 1958 ± 19 W, T2: 2139 ± 373 W; Hedges’ g: 0.577), 6 × 8 group (T1: 1979 ± 368 W, T2: 2123 ± 432 W; Hedges’ g: 0.380), and 8 × 8 group (T1: 2082 ± 512 W, T2: 2197 ± 466 W; Hedges’ g: 0.577), respectively, with no significant differences among groups (Group, Time × Group Interaction: p > 0.05).

**Figure 2:**
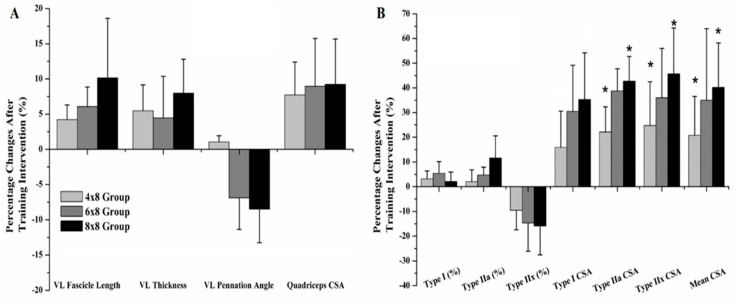
Percentage changes after 5 weeks of training with fast eccentric-only half squats in each group for (**A**) Vastus Lateralis (VL) architecture properties and quadriceps cross sectional area (CSA) and (**B**) Vastus Lateralis muscle fiber composition. * = significant differences between the marked group in each parameter.

Vastus lateralis fascicle length and pennation angle were altered significantly (Factor Time: p < 0.001; η^2^: 0.226–0.567; Table 2) in 6 × 8 group and 8 × 8 group (Figure 2A), with no significant difference between them (Group, Time × Group Interaction: p > 0.05). Vastus lateralis thickness was not changed in any group (Factor Time, Group, Time × Group Interaction: p > 0.05). Quadriceps cross sectional area was increased significantly in all groups (p < 0.01; η^2^: 0.606; Table 1), with no significant differences among the groups (Group, Time × Group Interaction: p > 0.05).

Vastus lateralis muscle fiber composition was not altered after the training in any of the groups (Factor Time: p > 0.05; Table 3, Figure 2B). However, it must be noted that a non-significant reduction of the percent type IIX muscle fibers was found in all groups, with the highest reduction to observed in the 8 × 8 and the lowest in the 4 × 8 group (Table 3). The CSA of all fiber types was increased significantly, in all groups, with the highest increases found in the 8 × 8 group and the lowest in the 4 × 8 group (Factor Time: p < 0.001; η^2^: 0.185–0.808; Table 3, Figure 2B). Fiber CSA at T2 was significantly different between 4 × 8 and 8 × 8 groups (Time × Group Interaction: p < 0.05; Table 2). Likewise, the percent changes of the CSA of type IIa and IIX fibers, as well as the mean CSA (all fibers calculated together) were significantly different between 4 × 8 and 8 × 8 groups (Time × Group Interaction: p < 0.05; Table 3) after the training. No significant correlations were observed between the changes in performance and the changes of the biological parameters, in response to the training stimulus (p > 0.05).

## 4. Discussion

The main finding of the present study was that four sets with eight repetitions of fast eccentric half-squats performed twice per week are adequate to increase lower body muscle strength and power during the initial 5 weeks of training in moderately trained females. It seems that two to three times larger training volumes than 4 × 8 twice a week, of this exercise, may not result in larger increases in explosive performance, during the initial 5 weeks of training, however, performing increased number of repetitions per training session result to higher increases of muscle mass. Interestingly, the changes of VL muscle fiber composition, thickness, fascicle length, pennation angle and the quadriceps cross sectional area could not explain the changes in power performance induced by either of the training volumes. Therefore, these physiological variables may not provide a direct explanation of the performance increase during the initial phase of fast eccentric training in these previously untrained females. Previous studies report that resistance training-induced increases in muscle mass, have been linked with increases in explosive performance [7,27,29]. Here, quadriceps mass and VL muscle fiber cross sectional area were significantly increased in all groups, with the larger training volume inducing larger increases, while VL fascicle length was increased in 6 × 8 and 8 × 8 but not in 4 × 8 group. Yet, jumping performance increased similarly in all groups. Therefore, it seems that other physiological mechanisms, such as neural adaptations, may have contributed to power performance enhancement, besides quadriceps mass hypertrophy, architecture and fiber type composition. The role of neural drive in explosive performance has been described in the past, with more recent studies emphasizing the role of maximal motor unit discharge rate in explosive actions [22,44]. Regardless of the physiological mechanism supporting performance enhancement, the current results may suggest that in moderately trained females, a low volume training plan, including only 32 fast eccentric half-squats twice a week, may result in significant increments in lower body explosive performance, after a few weeks of implementation. 

Interestingly, neither of the training volumes used here, induced a significant reduction in vastus lateralis type IIX muscle fiber percentage. This is in accordance with previous studies reporting that power training may preserve the proportion of type IIX muscle fibers, at least in sedentary or moderate trained participants during the initial weeks of explosive training [3,7,9,10,12,23,24,25,32]. Significant reductions in type IIX muscle fiber percentage with power training have been reported before [45]. However, in that previous study, the training volume was significant higher (80–180 muscle actions) compared to these used in the present study (30–64 muscle actions per training session). Indeed, the majority of studies reporting maintenance of type IIX muscle fibers, after power training, have used relatively low training volumes, e.g., lower than 80 muscle contractions per training session [3,7,9,10,12,24,25,32,46], as used in the present study. However, the length of the training procedure of the present study, may have a role in the non-significant reduction of type IIx muscle fibers percentage. Maybe, a longer training duration combined with larger training volumes may induce a significant reduction of these muscle fibers. 

Vastus lateralis fascicle length increased only after 6 × 8 and 8 × 8 of fast eccentric-only squats. Fascicle length is thought to reflect the number of sarcomeres in series in a muscle fascicle [47]. An increased number of sarcomeres in series results in greater fascicle shortening velocities, which may be associated to higher power production [48]. However, in the present study, fascicle length increased only after 6 × 8 and 8 × 8, while power performance increased similarly in all groups including the 4 × 8 group. In addition, no correlations were found between the training induced changes in fascicle length and those of power performance. These observations indicate that VL fascicle length increases may not be directly linked to changes in explosive performance during the initial phase of power training. In previous studies, significant increases in VL fascicle length have been reported in response to fast eccentric power training [11,14,25]. Moreover, a significant correlation has been reported between the training induced changes in VL fascicle length and changes in leg press isometric rate of force development [11]. However, the training volume was larger, and the training duration was longer in that previous study, compared with the current study. The current results suggest that a relatively large fast eccentric-only squats training volume is necessary for vastus lateralis fascicle length changes to occur.

Resistance training elicits significant neural adaptations, especially during the first training weeks [28]. Accordingly, it is anticipated that neural adaptations might have a major role in power performance increases that observed in the current study, yet unfortunately, such adaptations were not evaluated. Another limitation of the present study was the relative short training period. Perhaps a longer training period could have induced a training volume dose-response relationship effect on explosive performance and on the biological parameters investigated in the present study. Nevertheless, it was the aim of the study to investigate changes in explosive performance after such a short intervention with eccentric squats. Training induced adaptations of other lower extremities’ muscles (like the triceps surae) which are heavily involved in lower body power performance were not evaluated here, and this might have provided a better insight into the training induced adaptations and their relationship to performance enhancement. Finally, a limitation of the present study was that the present participants had minimal resistance training history prior to their entry in the study. However, the present study aimed to recruit moderate trained female participants, with no recent background in systematic resistance training programs. Thus, future studies should investigate the possible dose-response relationships between power training volumes and training induced adaptations on muscle power/strength and muscle morphology, in trained male and female participants.

## 5. Conclusions

In conclusion, the current data suggest that 64 repetitions per week of fast eccentric half-squat training are sufficient to increase lower body muscle strength and power, after 5 weeks of training, in moderately trained females. This training regimen induces significant neuromuscular adaptations including increases in muscle fiber cross sectional area, muscle architectural changes, and maintenance of type IIX muscle fibers. Although not measured here, neural adaptations may have also contributed significantly to the increase in explosive performance.

## Figures and Tables

**Table 1:** Basic anthropometric characteristics of the three groups of participants. No significant differences were found among the groups (p > 0.05).

Group	Age (Years)	Mass (kg)	Height (cm)	Body Mass Index (kg·m^−2^)
**4 × 8 (n = 9)**	20.8 ± 2.7	56.7 ± 6.7	163.3 ± 6.6	21.3 ± 2.1
**6 × 8 (n = 8)**	21.6 ± 2.3	59.1 ± 10.7	165.1 ± 6.0	21.6 ± 3.1
**8 × 8 (n = 8)**	21.4 ± 2.4	58.4 ± 5.9	167.1 ± 7.3	22.5 ± 2.5

**Table 2:** Vastus lateralis’ architecture morphology and quadriceps cross sectional area before and after 5 weeks of training for each group.

	6 × 8 Group (N = 8)				8 × 8 Group (N = 18)								Partial η^2^			
	Pre	Post	Hedges’ g	%	Pre	Post	Hedges’ g	%					Time	Group	Time X Group	
**Vastus Lateralis’ Architecture Morphology**																
**Fascicle Length (cm)**	7.69 ± 0.79	8.04 ± 1.13	0.375	4.22 ± 2.09	7.57 ± 0.78	8.02 ± 0.67 *	0.644	6.06 ± 2.80	7.52 ± 0.65	8.26 ± 0.69 *	1.162	10.15 ± 8.46	0.567	0.003	0.123	
**Thickness (cm)**	2.09 ± 0.47	2.19 ± 0.49	0.233	5.46 ± 3.68	2.28 ± 0.45	2.37 ± 0.36	0.214	4.46 ± 5.92	2.25 ± 0.42	2.31 ± 0.41	0.145	7.96 ± 4.84	0.190	0.037	0.014	
**Pennation Angle (^o^)**	18.04 ± 6.06	18.12 ± 5.68	0.014	1.05 ± 0.89	19.33 ± 5.00	17.62 ± 4.28 *	−0.389	−6.90 ± 4.47	18.01 ± 3.82	16.33 ± 3.12 *	−0.512	− 8.49 ± 5.23	0.226	0.016	0.143	
**Quadriceps Cross Sectional Area**																
**Cross Sectional Area (cm^2^)**	48.71 ± 6.73	52.05 ± 4.66 *	0.637	7.71 ± 4.69	51.32 ± 7.66	55.56 ± 6.36 *	0.641	8.95 ± 6.80	56.14 ± 11.08	60.44 ± 10.41 *	0.425	9.22 ± 6.45	0.606	0.164	0.019	

Values are represented as Mean±SD. With (*) denoted the significant differences between pre and post values in each group. P < 0.05.

**Table 3:** Vastus lateralis’ muscle fiber composition before and after 5 weeks of training for each group.

	4 × 8 Group (N = 9)				6 × 8 Group (N = 8)				8 × 8 Group (N = 8)				Partial η^2^		
	Pre	Post	Hedges’ g	Percentage Change	Pre	Post	Hedges’ g	Percentage Change	Pre	Post	Hedges’ g	Percentage Change	Time	Group	Time X Group
**Type I (%)**	46.96 ± 6.49	48.03 ± 5.86	0.110	3.17 ± 3.2	49.43 ± 4.43	51.92 ± 4.45	0.165	5.45 ± 4.7	49.52 ± 9.73	49.66 ± 7.39	0.017	2.12±3.80	0.041	0.054	0.024
**Type IIa (%)**	42.81 ± 7.50	43.33 ± 5.90	0.086	2.02 ± 4.79	40.60 ± 3.60	42.51 ± 5.12	0.133	4.70 ± 3.22	40.49 ± 8.3	44.81 ± 9.96	0.198	11.57 ± 9.41	0.174	0.009	0.162
**Type IIX (%)**	10.23 ± 3.52	9.26 ± 3.52	−0.033	−9.56 ± 7.88	9.02 ± 1.95	7.74 ± 1.72	−0.191	−14.67 ± 11.42	10.23 ± 2.87	8.62 ± 2.71	−0.215	−15.89 ± 11.68	0.041	0.046	0.024
**Type I CSA (μm^2^)**	3198 ± 604	3683 ± 1059	0.622	15.89 ± 14.68	3270 ± 416	4145 ± 452 *	1.899	30.41 ± 25.71	3266 ± 792	4308 ± 525 *	2.026	35.28 ± 18.90	0.545	0.073	0.098
**Type IIa CSA (μm^2^)**	3624 ± 650	4148 ± 729 *	0.840	22.09 ± 10.25 #	3505 ± 450	4482 ± 537 *	1.650	38.77 ± 9.07	3650 ± 709	4791 ± 683 *	1.985	42.74 ± 10.09 #	0.749	0.063	0.221
**Type IIX CSA (μm^2^)**	2692 ± 513	3333 ± 581 *#	1.296	24.74 ± 14.75 #	2684 ± 381	3813 ± 361 *	2.870	36.15 ± 20.64	2605 ± 520	4024 ± 474 *#	3.059	45.64 ± 18.65 #	0.808	0.114	0.290
**Mean CSA (μm^2^)**	3309 ± 548	3979 ± 723 *	1.156	20.77 ± 15.79 #	3279 ± 387	4349 ± 418 *	2.190	35.36 ± 29.87	3367 ± 629	4840 ± 537 *	2.674	40.20 ± 18.62 #	0.773	0.099	0.185

Values are represented as Mean ± SD. With (*) denoted the significant differences between pre and post values in each group, while with (#) and ($) denoted the significant differences between the marked variables. CSA = Cross Sectional Area. P < 0.05.
